# Study of CEO Narcissism and Corporate R&D Investment

**DOI:** 10.3389/fpsyg.2022.888618

**Published:** 2022-05-13

**Authors:** Linan Wang, Huiying Li, Yinghan Mu

**Affiliations:** School of Accounting, Jilin University of Finance and Economics, Changchun, China

**Keywords:** upper echelons theory, CEO narcissism, R&D investment, financing constraints, nature of ownership

## Abstract

Narcissism is a deep-rooted personality trait that is particularly common in corporate leaders, and narcissistic leaders have a noticeable influence on the R&D investment behavior of enterprises. This paper empirically examines the effect of CEO narcissism on R&D investment and the moderating effects of financing constraints, and the nature of corporate ownership based on the Upper Echelons Theory, using the CEO signature size as a measure of CEO narcissism for the 2007–2020 Chinese A-share listed companies. The results show that CEO narcissism has a significant negative effect on R&D investment; corporate financing constraints play a negative moderating role between CEO narcissism and R&D investment, and the negative effect of CEO narcissism on corporate R&D investment is more significant in non-state-owned firms than in state-owned firms. The study’s findings enrich and expand the theory related to CEO narcissism and have important practical implications for R&D investment decisions and the choice of corporate executives in China.

## Introduction

In today’s globalized economy and rapidly changing technologic world, innovation is a key source for enterprises to gain and maintain competitive advantage, and a fundamental driver of a country or region’s economic growth. R&D investment is a key input for enterprise innovation, which can improve the technological innovation capability, reduce production costs, and accelerate the process of new product development, thus helping enterprises to establish and maintain a competitive advantage (Wang et al., 2017), therefore increasing R&D expenditure is the foundation and prerequisite for improving innovation capability. Accordingly, investigating which factors influence R&D investment is a question worthy of deeper analysis, as internal factors, especially the management dimension, play a more decisive role in R&D investment decisions than external factors (Griffiths and Webster, 2010).

With the increasing complexity of the corporate decision-making environment and the development of upper echelons theory, society and academia are becoming increasingly aware of the important role of top managers in corporate growth. As the core of the top management team, the capabilities and attributes of the CEO will influence the quality of corporate decisions, affect the business results, and even determine the success or failure of the company. Upper echelons theory suggests that managers’ experience, values and personal characteristics have an important role in corporate decision-making (Hambrick and Mason, 1984; Hambrick, 2007). Managers are independent and different from each other based on their attributes. CEO age, CEO tenure, and CEO educational background have an important influence on managers’ strategic decision-making influence, which in turn affects healthy development (Gupta et al., 2018; Gupta, 2021, 2022). Abatecola and Cristofaro (2019) focus on the impact of CEO attributes, namely CEO power, CEO personality, CEO profifiles and CEO effect, on sustainable behavior, pointing out that narcissism and arrogance play a key role in strategic dynamic rapid decision-making and efficient communication, and have a negative impact on CEO sustainable behavior. Naheed et al. (2021) demonstrated a significant relationship between managerial capacity and firm investment, which is more prominent in firms with small financing constraints and good financial conditions. Farag and Mallin’s (2018) studies found that younger and shorter tenured CEOs and those with postgraduate qualifications are more likely to consider risky decisions. At present, domestic and foreign scholars have conducted preliminary research on the relationship between managerial personality traits, CEO narcissism, and entrepreneurial behavior, mainly based on the upper echelons theory. Maliik and Kousar (2019) used CEO narcissism as a moderating variable and found that CEO narcissism remarkably moderated the relationship between founder CEO and entrepreneurial orientation and had no significant effect on the relationship between CEO ownership and entrepreneurial orientation. The study results by Chatterjee and Hambrick (2007) showed that CEOs with narcissistic tendencies are more likely to cause major changes in corporate strategy. Narcissistic CEOs are more willing to take risks, and they will subjectively assign a high probability of success to investment projects with high risk and high return characteristics when making investment project decisions, and this tendency to prefer high-risk and high-return investment projects will lead to a greater likelihood of changing radically in firm performance (Zhu and Chen, 2014). CEOs with high levels of narcissism disguise their company’s performance by increasing profits through the avoidance of taxes in order to exaggerate corporate value (Olsen and Stekelberg, 2016). The higher the level of narcissism, the higher the probability that the CEO will commit corporate financial fraud to exaggerate the value of the firm and improve his or her positive social image (Olsen et al., 2014).

It can be seen that the existing studies on CEO narcissism are still fragmented, one-sided and have not yet reached a consensus, and the relevant studies mainly focus on CEO narcissism itself. Although a number of studies on R&D investment have been discussed from the perspective of psychology, there are few studies on narcissism in the existing literature, and the influence mechanism between the degree of CEO narcissism and corporate R&D investment decisions is rarely addressed, without considering the financing constraints or ownership nature of the contextual factors and their roles and functions in the path of CEO narcissism on corporate R&D investment. There is still a gap in theoretical research. Therefore, it is necessary to study CEO narcissism, corporate R&D investment, financing constraints, and ownership nature under the same framework. Moreover, with the rapid socio-economic development and the interpenetration of Eastern and Western cultural values, people’s narcissism level has been increasing. In fact, there are some CEOs with obvious narcissistic personality traits in both China and foreign countries who have driven their companies to break through technological barriers and achieve corporate success. Therefore, it is of great theoretical and practical significance to study the relationship between CEO narcissism and business, both at the academic and practical levels. Therefore, on the basis of the existing studies, this paper takes the A-share listed companies in China from 2007 to 2020 as the research sample, adds the financing constraints and the nature of enterprise ownership into the research framework, and tries to explore the following issues: (1) As the top decision-maker of the enterprise, does the degree of the narcissism of the CEO affect the R&D investment decisions of the enterprise? (2) Do financing constraints and the nature of ownership affect the relationship between CEO narcissism and corporate R&D investment? How do they affect it? The possible research contributions of this paper are as follows: First, the perspective of executive personality traits is chosen to study the issue of corporate R&D investment behavior. Unlike most previous studies on overconfidence traits, this paper explores the impact of executive narcissism traits on firms, which broadens the research perspective of existing corporate investment theories, using financing constraints and ownership nature as moderating variables to study their effects on CEO narcissism and R&D investment, which enriches the literature on CEO narcissism. Second, based on existing foreign studies and weighing the availability of data, we use CEO handwritten signature size to measure leader narcissism, introducing a new approach to the narcissism measurement system and providing a reference for similar studies in the future.

## Theoretical Analysis and Research Hypothesis

### CEO Narcissism and Signature Characteristics

With the increasing number of individuals with narcissistic personality traits in organizations, scholars in the fields of management and organizational behavior have begun to include narcissistic personality traits in their research. First, the psychological traits associated with narcissists include authority, superiority, possessiveness, entitlement, vanity, and conceit (Raskin and Terry, 1988), and a core component of narcissism as a personality trait is a unique and superior self-perception (Emmons, 1987). Second, narcissism is a kind of innate and relatively stable personality trait that is ubiquitous in people (Cramer, 1998), and changes in the external environment and other objective conditions have little impact on it (Raskin and Terry, 1988; Campbell et al., 2004). For narcissistic company executives, no matter whether the company’s performance is good or bad, or the social evaluation is high or low, he will strive to pursue and shape his own superiority and perfect image (Stolorow, 1975). Narcissism is a particularly common personality trait in top executives, such as CEOs. Narcissistic leadership is generally viewed as a negative leadership trait because the behavior of narcissistic leaders is driven by their own personal needs rather than by organizational interests, and narcissistic leaders exercise their power primarily based on personal goals or self-fulfillment motives. When personal interests conflict with organizational interests, they seldom consider the organizations. Thus the negative impact of narcissistic leadership is greater than the positive impact (Rosenthal and Pittinsky, 2006). In contrast, Khoo and Burch (2008) argue that narcissistic leaders have both positive and negative aspects. They may have both negative effects and destructive effects, and they may bring good performance to the organization, especially when the environment is volatile. Narcissistic leaders’ decisive decisions, and their persistence in self-determination, often bring benefits to the organization. However, both the positive and negative effects of narcissism on the organization are significant for both ordinary narcissists in organizations and narcissists in leadership positions. Therefore, it is important to identify whether an individual in an organization is narcissistic and to measure the level of individual narcissism.

The measurement of the degree of narcissism in CEOs is a difficult and crucial aspect of studying its effects. The questionnaire measurement of narcissism has matured research results in academic research. At present, foreign scholars mostly use the NPI scale to measure narcissistic personality (Raskin and Hall, 1979). On this basis, Ames et al. developed a self-reported personality scale that includes 16 (Ames et al., 2006). However, the reliability of executives completing the NPI self-assessment questionnaire has been questioned, for narcissistic leaders often refuse to fill out or do not fill out the self-assessment questionnaire truthfully (Owens et al., 2015), and the practicality and reliability of his assessment questionnaire are difficult to ensure. In addition, the measurement methods using objective proxy variables, such as the size of CEO photos in the annual report, the frequency of the CEO using the first person in interviews, the CEO’s relative salary, and other indicators are not suitable for the Chinese context. Firstly, for the index of “using the first person,” some studies have pointed out that Chinese culture prefers “we” rather than “I” (Zhang, 2010), which makes it difficult to highlight the CEO’s personal awareness. Secondly, “relative CEO compensation” is also limited in China since the CEO compensation of most state-owned enterprises is not decided by the CEOs themselves but is controlled by the State-owned Assets Supervision and Administration Commission (Chen et al., 2009). Furthermore, after consulting the annual reports issued by the CSRC, we found that few listed companies use the CEO’s photo in their annual reports, which means the index of “CEO photo size in annual reports” is hard to apply in the Chinese context. If the above indicators are used to study narcissism among Chinese corporate leaders, there will be a large bias. Hence, we need to find other indicators to identify whether CEOs are narcissistic or not when choosing non-questionnaire measures. In recent years, due to a certain correlation between signature size and individual narcissism, signature size is used to measure an individual’s self, which does not require participants to answer questions about personality. Because participants may not know that their self characteristics affect something that is so simple, for example, a signature, individual signature size has become a way to measure narcissism, and scholars at home and abroad have carried out in-depth research on the individual signature size and narcissistic personality traits. Zweigenhaft (1970) and Zweigenhaft and Marlowe (1973) found that individuals with larger signatures tended to exaggerate their self-perceptions and exhibited strongly perceived narcissism. Snyder and Fromkin (1977) found that individuals with larger signatures had a sense of superiority, and Jorgenson (1977) found that people with larger signatures would tend to exhibit control and dominance over others, and Zweigenhaft (1977) further demonstrated that signature size could be used as an implicit indicator of ego and dominance, both of which are associated with narcissism. A growing number of studies have demonstrated a significant link between handwritten signatures and personality (Furnham and Gunter, 1987; King and Koehler, 2000), with signatures being strongly linked to self-identity and handwritten signatures being more reflective of self-awareness (Bouletreau et al., 1998) and better demonstrating an individual’s identification with themselves. As a presentation of self-identity, the handwritten signature is a medium for projecting self-awareness and reflects the individual’s superior self-perception (Kettle and Häubl, 2011). However, a positive correlation between signature size and self-esteem, self-awareness, and self-identity does not guarantee a positive correlation between signature size and narcissism. Therefore, Ham et al. (2018) validated signature size as a measure of narcissism through a laboratory study using phonetic script samples to experimentally demonstrate a positive correlation between signature size and narcissism, providing evidence for the use of signature size as a measure of narcissism level method to provide practical experience and evidence.

### CEO Narcissism and R&D Investment

Due to narcissists’ craving for power and seeking authority, narcissistic CEOs need to consistently gain the attention, applause, and admiration of others and thus maintain their exaggerated image (Gerstner et al., 2013). To gain recognition and applause from others, narcissistic CEOs tend to create and implement situations that can attract the attention of others (Wales et al., 2013). Chatterjee and Hambrick (2011) found that CEO narcissism was significantly and positively related to both the size and number of mergers and acquisitions of the firm; Judd et al. (2017) showed that the higher the level of the narcissism of acquirers’ CEOs, the higher the likelihood they will implement M&A actions to satisfy their own psychological need for attention through such behavior, while narcissistic CEOs will place more emphasis on short-term financial control rather than long-term strategic synergy (Hitt et al., 1990); thus enhancing the probability of M&A failure. The performance pressure caused by the failure of M&A may cause managers to reduce the R&D investment of enterprises, weaken the process of promoting new products and processes within the company, and even lead to the loss of core research teams, which may even harm the innovation ability of enterprises (Hitt et al., 1991). Therefore, the narcissistic leader’s vanity and popularity characteristics make him/her more eager to pursue scale and have the motivation and tendency to build a “business empire” (Raskin and Terry, 1988). This strategy of external expansion will reduce the firm’s expenditure on R&D.

Narcissists’ superiority and exploitative nature make them more likely to invest based on their personal preferences and interests. Thus, on the one hand, narcissistic CEOs will be more superior and privileged, and they have a higher likelihood of being stubbornly unwilling to make strategic changes out of overconfidence in the outcome of their decisions, believing it could be optimal for the company’s development. On the other hand, narcissists’ conceited characteristics encourage them to increase their optimistic expectations for the future, that is, to overestimate the benefits brought by investment projects and underestimate the risks attached to the investment. Ham et al. (2018) argue that narcissistic CEOs implement more R&D and M&A investments, while narcissistic CEOs tend to pursue an investment style with lower returns because of the lower profitability of the investment project and lower operating cash flows. Therefore, even if initially firms make R&D investments, CEOs will stop investing funds due to the high uncertainty of economic returns or blind investments leading to R&D failures, which eventually results in much lower R&D investments and, at the same time, greatly reduces CEO R&D investment incentives. As a result, the following hypothesis is proposed.

H1: CEO narcissism is negatively correlated with corporate R&D investment.

### The Moderating Effect of Financing Constraints

Although narcissism and overconfidence are different psychological traits, narcissistic CEOs tend to exhibit overconfidence, and both have similar effects on corporate investment and financing behaviors. In terms of investment behavior, in 1986, Roll proposed the classic “arrogance hypothesis,” which is characterized by managerial overconfidence, suggesting that managerial overconfidence may lead to excessive takeover activities and stimulate more takeovers (Roll, 1986). Overconfident CEOs will overestimate their ability to generate profits in their existing firms and potential takeover targets and will believe that outside investors underestimate the actual value of their existing firms, so overconfident CEOs are likely to make value-losing acquisition decisions (Malmendier and Tate, 2005). When free cash flow exists, over-optimistic managers overestimate the net present value of their investment projects, leading to over-investment (Heaton, 2005). In terms of financing behavior, overconfident managers choose higher debt financing and issue new debt at a higher frequency, leading to a shorter maturity structure of debt (Hackbarth, 2008). Entrepreneurs’ optimistic expectation bias has a fairly robust positive relationship with the use of short-term debt, and this optimistic expectation bias will persist and have a significant impact on the firm’s capital structure (Landier and Thesmar, 2008). Therefore, overconfident or narcissistic managers can lead firms to make aggressive investment and financing decisions.

The financing constraint theory argues that, in reality, the external financing cost of enterprises is much higher than the internal financing cost due to the existence of agency costs and information asymmetry. Enterprise innovation investment requires continuous capital investment and high demand for financing. Thus it is vulnerable to the influence of financing constraints. In general, the existence of financing constraints limits the source of funds for enterprises, and if internal resources are insufficient, enterprises will give up investing in larger innovation projects, resulting in insufficient R&D investment; the existence of financing constraints increases the possibility of R&D investment failure, thus enhancing managers’ risk-averse tendencies. However, because narcissistic leaders are more confident and base their decisions primarily on expectations rather than actual performance, narcissistic leaders will tend to choose riskier strategies (Campbell et al., 2004); a family firm decision-maker who is highly narcissistic will not tend to choose conservative, risk-averse strategies when formulating business development plans (Jones et al., 2008). Consequently, narcissistic CEOs lead firms to adopt more aggressive financing decisions, and narcissistic CEOs do not reduce their investment in R&D even when firms face financing constraints. The greater the financing constraint, the lower the disincentive effect of CEO narcissism on R&D investment. The degree of financing constraints varies by firm size. Gertler and Gilchrist. (1993) state that smaller firms have more external finance premium than larger firms, which could be due to two reasons: first, large firms have more collateral assets that help them to finance their investments easily; and secondly, large firms might be having their business group that helps them to use their own internal capital market. Gertler and Gilchrist (1994) argued that small companies act as proxies for financially constrained firms because these companies exhibit greater bank dependencies, cannot issue public debt, and face a higher level of Apart from this, smaller firms are usually younger, with a high level of firm-specific risk and less collateral, thereby reducing the possibility of attracting external finance. Gupta et al. (2021) also documented that small firms are more financially constrained than large firms. Compared to small firms, large firms have abundant access to finance and lower financing costs, and R&D activities require continuous corporate investment, so CEOs of large firms have more continuous motivation and strength to invest in R&D. Accordingly, this paper proposes the following hypothesis.

H2a: Other things being equal, financing constraints negatively moderate the relationship between CEO narcissism and R&D investment.

H2b: Financing constraints play a significant moderating role between CEO narcissism and corporate R&D investment in large firms compared to small firms.

### The Moderating Effect of the Nature of Ownership

Corporate ownership can play a decisive role in the internal governance arrangements of a firm, fundamentally determine the way resources are allocated, and thus profoundly influence corporate R&D investment behavior. Upper echelons theory suggests that the extent to which individual executive traits influence corporate decisions varies by context (Bromiley and Rau, 2016). Managerial Discretion is an important moderating variable (Hambrick, 2007), and whether top managers have managerial autonomy is a core criterion for distinguishing strategic management from institutional and competitive schools of thought; the more managerial autonomy top managers have, the greater their influence on corporate strategic decisions. In recent years in China, private enterprises have created an increasing share of economic returns in the national economy. Because their organizational structure and ownership nature are very distinguished from those of state-owned enterprises, the enterprises are relatively less constrained by government politics. To a certain extent, the private enterprise CEO has greater management autonomy and can make independent decisions on R & D investment required by enterprise innovation activities.

On the one hand, SOEs have abundant external controllers and are mostly owned by the state or local SASACs, which can provide reliable financial support for innovation. This can reduce the risk of uncertainty due to institutional and policy changes and the uncertainty of the external environment, hence facilitating innovation (Choi et al., 2011). While private enterprises have relatively unstable funding sources, top managers will reduce their R&D investment. At the same time, along with the crowding-out effect of state-owned enterprises’ R&D investment on private enterprises’ R&D investment, the gap between state-owned and non-state-owned enterprises’ innovation performance will further increase so that state-owned enterprises can concentrate on more high-quality innovation resources and R&D funds, as well as have a stronger innovation capability. In contrast, private enterprises are just the opposite. Therefore, private CEOs are less likely to increase their R&D investment. On the other hand, narcissistic CEOs are eager for others’ attention and praise and likewise, have a strong desire for power control. Since private enterprise CEOs originally had greater management autonomy, so narcissistic private enterprise CEOs are eager to achieve great results in the short term to obtain job promotion and external attention. However, it takes a long time to achieve innovative R&D results, and private enterprise CEOs with a high degree of narcissism only focus on their immediate interests. They are not willing to make long-term and unpredictable R&D investments. Based on this, this paper proposes the following hypothesis.

H3: The negative effect of CEO narcissism on corporate R&D investment is more significant in non-state-owned firms compared to state-owned firms.

**FIGURE 1:**
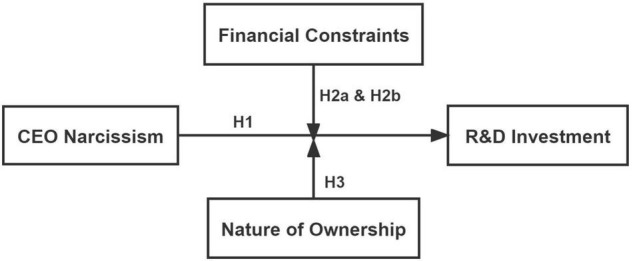
Research framework.

Based on the above analysis, the research framework constructed in this paper is shown in Figure 1.

## Research Design

### Sample Selection and Data Sources

China’s A-share listed companies started to implement the new Accounting Standards for Business Enterprises (ASBE) in 2007. In order to reduce the influence of the institutional environment on the results, this paper selects listed companies in Shanghai and Shenzhen from 2007 to 2020 as the initial research sample and screens the sample according to the following conditions: (1) exclude listed companies in finance and insurance; (2) exclude listed companies with a trading status of ST and *ST in that year; (3) exclude listed companies with narcissistic CEO and incomplete or missing data of major financial indicators. The final valid sample of 1282 observations is obtained, and all continuous variables are winsorized above and below 1% in order to prevent the influence of extreme values. CEO signature data are collected and collated by hand and processed by Python, mainly from IPO prospectuses, and other related data are obtained from the CSMAR database.

### Definition and Measurement of Variables

#### CEO Narcissism

CEO narcissism, the independent variable in this study, is a stable and prevalent personality trait in which CEOs focus attention on themselves and overrate themselves. Research in the field of psychology has shown that signature size has a positive correlation with narcissism. Drawing on Ham et al. (2017, 2018), CEO signature size is used to measure the degree of CEO narcissism, and the larger its value, the higher the degree of CEO narcissism. Based on the unique institutional context in China, this paper identifies the research object as the CEO or chairman of a listed company and uses Python programming to locate the smallest rectangle occupied by the handwritten signature of the CEO in the IPO prospectus of a listed company, and obtains the number of pixels within the rectangle through the feedback of the program to determine the signature size. In order to eliminate the effect of word count on area, this paper normalizes by dividing by the number of signature words in order to obtain the proxy variable for CEO narcissism finally.

#### R&D Investment

Referring to the method of measuring R&D investment by Yoo and Rhee (2013), it is expressed by the annual R&D investment ratio to total assets at the end of the period.

#### Financing Constraints

The financing constraint is a significant difference between the cost of internal financing and the cost of external financing of the firm (Fazzari et al., 1987). Based on Fee et al. (2009), this paper selects several financial indicators representing the company’s operating status to construct the Logit model to measure the degree of financing constraints of the company. First, the three variables of firm size, age, and cash dividend payout ratio are standardized by year, and the dummy variable of financing constraint QUFC is determined by ranking the mean values of the variables after standardization, with listed firms greater than 66% quantile defined as low financing constraint group with QUFC = 0, and those less than 33% quantile defined as a high financing constraint group with QUFC = 1. Second, a logit model is used for regression to fit the probability of occurrence of a financing constraint for each year of the firm, and it is defined as the financing constraint index FC (taking values between 0 and 1). The larger the FC, the more serious the financing constraint problem of the firm. In model (2), Lev denotes asset-liability ratio, Cashdiv denotes cash dividends declared in the year, Ta denotes total assets, MB denotes market-to-book ratio, NWC denotes net working capital, and EBIT denotes earnings before interest and taxes.


(1) $$P\left(QUFC=1|Z_{i,t}\right)=e^{Z_{i,t}}/\left(1+e^{Z_{i,t}}\right)$$

(2) $$Z_{i,t}=\alpha_{0}+\alpha_{1}\,S\,i\,z\,e_{i,t}+\alpha_{2}\,L\,e\,v_{i,t}+\alpha_{3}\,{\left(C\,a\,s\,h\,d\,i\,v/T\,a\right)}_{i,t}+\alpha_{4}\,M\,B_{i,t}+\alpha_{5}\,{\left(N\,W\,C/T\,a\right)}_{i,t}+\alpha_{6}\,{\left(E\,B\,I\,T/T\,a\right)}_{i,t}$$

#### Nature of Business Ownership

The nature of ownership as a dummy variable, when the actual controller of the enterprise is the state or the institutions and institutions representing the state, the value is 1. Otherwise, the value is 0.

#### Control Variables

In this paper, control variables are introduced mainly at the firm financial level, corporate governance level, and CEO’s own characteristics level. The control variables at the financial level include return on total assets (Roa), asset-liability ratio (Lev), market-to-book ratio (MTB), cash holdings (CF), and firm size (Size). The control variables at the corporate governance level include the percentage of independent directors (Indir) and dual positions (Dual). The control variables at the CEO’s own characteristics level include CEO gender (Gender), CEO age (Age), CEO education (Degree), and CEO overseas background (Oversee), and also control for year and industry fixed effects. The above variables and explanations are shown in Table 1.

**TABLE 1:** Definition and measurement of variables.

Variable type	Variable name	Symbol	Measurement method
Dependent variable	Investment in R&D	RD	R&D investment/operating income
Independent variable	CEO Narcissism	Nar	Ln (number of pixels occupied by the signature)
Moderating variables	Financing constraints	FC	Calculated from models (1) and (2)
	Nature of business ownership	Soe	State-owned enterprises = 1, non-state-owned enterprises = 0
Control variables	Return on total assets	Roa	Net profit/total assets
	Asset-liability ratio	Lev	Total liabilities/total assets
	Book-to-market ratio	MTB	Shareholders’ equity/company market capitalization
	Cash holdings	CF	Operating cash flow/total assets
	Company size	Size	Ln (total assets)
	Proportion of independent directors	Indir	Proportion of independent directors
	Two jobs in one	Dual	Chairman and Managing Director are the same as 1, otherwise 0
	CEO Gender	Gender	Male = 1, Female = 0
	Age of CEO	Age	Actual age in the year
	CEO qualifications	Degree	≤ Associate degree = 1, Bachelor = 2, Master = 3, Doctor = 4
	CEO Overseas Background	Oversee	1 if the CEO has overseas study or employment experience, 0 otherwise

### Model Construction

To test the research hypothesis of this paper, the following model was constructed for this study.


(3) $$R\,D=\alpha_{0}+\alpha_{1}\,N\,a\,r+\sum \alpha \,C\,o\,n\,t\,r\,o\,l\,s+\varepsilon$$

(4) $$R\,D=\beta_{0}+\beta_{1}\,N\,a\,r+\beta_{2}\,F\,C+\beta_{3}\,N\,a\,r*F\,C+\sum \beta \,C\,o\,n\,t\,r\,o\,l\,s+\delta$$

Among them, Nar × FC denotes the interaction term between CEO narcissism and the nature of firm ownership, and Controls denote the control variables. Model (3) is used to test the effect of CEO narcissism on R&D investment, and model (4) is used to test the moderating role of financing constraints between CEO narcissism and R&D investment. In order to test the moderating effect of financing constraints between large and small companies, the sample companies are ranked according to their operating income and listed companies larger than 66% quantile are defined as large companies. In comparison, listed companies smaller than 66% quantile are defined as small companies, and model (4) is used to test the grouping of large and small companies, respectively. In order to test the moderating effect of the nature of enterprise ownership, this paper uses model (3) to test the grouping of state-owned enterprises and non-state-owned enterprises separately.

## Empirical Results and Analysis

### Descriptive Statistics

Table 2 reports the results of descriptive statistics for the main variables. As shown in Table 2, the mean value of R&D investment (RD) is 0.025, the minimum value is 0, and the maximum value is 0.108, indicating that there are significant differences in R&D investment among the sample companies. According to the Oslo Manual issued by the Organization for Economic Cooperation and Development (OECD), a company’s R&D investment intensity of 1 to 4 percent is considered to be medium. In terms of this standard, Chinese enterprises’ R&D and innovation capacity are basically at the middle level, with a certain R&D scale. The minimum value of CEO narcissism (Nar) is 6.418, the average value is 6.802, and the maximum value is 7.520. The minimum pixel of an executive signature is 612.82, and the maximum is 1845.10, which indicates that the degree of CEO narcissism varies among different sample companies. The minimum value of the financing constraint (FC) is 0.004, and the maximum value is 0.920. The average value of company attributes (Soe) is 0.203, indicating that about 20% of the companies are state-owned enterprises. More than nine out of 10 CEOs are male, and only one in 10 has an overseas background. The average age of the CEOs is 50.070 years old. When the personality trait attributes have taken shape, the relatively stable psychological state lays a solid foundation for the test of this paper.

**TABLE 2:** Descriptive statistical results of variables.

	No. of observation	Mean	SD	Min	Max
RD	1,282	0.025	0.021	0.000	0.108
Nar	1,282	6.802	0.202	6.418	7.520
FC	1,282	0.463	0.272	0.004	0.920
Soe	1,282	0.203	0.402	0	1
Roa	1,282	0.037	0.069	−0.290	0.195
Lev	1,282	0.409	0.178	0.060	0.811
MTB	1,282	0.351	0.160	0.077	0.817
CF	1,282	0.051	0.064	−0.115	0.238
Size	1,282	22.450	1.385	20.410	27.320
Dual	1,282	0.335	0.472	0	1
Indir	1,282	0.381	0.056	0.333	0.571
Gender	1,282	0.949	0.221	0	1
Age	1,282	50.070	7.276	27	82
Degree	1,282	2.310	1.202	0	4
Oversee	1,282	0.100	0.300	0	1

### Correlation Analysis

Table 3 shows the Pearson test for the variables. The results show that CEO narcissism, financing constraints, and R&D investment pass the Pearson correlation test. Furthermore, the absolute value of the correlation coefficient between the explanatory variables and the control variables is less than 0.5, indicating that there is no serious multicollinearity between the variables.

**TABLE 3:** Correlation analysis results.

	1	2	3	4	5	6	7	8	9	10	11	12	13	14	15
1.RD	1														
2.Nar	−0.097***	1													
3.FC	0.154***	–0.0210	1												
4.Soe	−0.139***	0.121***	−0.414***	1											
5.Roa	0.129***	−0.111***	0.025	–0.032	1										
6.Lev	−0.182***	0.095***	−0.583***	0.309***	−0.342***	1									
7.MTB	−0.185***	–0.036	0.214***	−0.124***	0.029	−0.461***	1								
8.CF	0.094***	−0.057**	−0.118***	−0.050*	0.394***	−0.198***	–0.017	1							
9.Size	−0.204***	0.023	−0.857***	0.506***	–0.006	0.537***	−0.073***	0.086***	1						
10.Dual	0.175***	−0.060**	0.160***	−0.226***	0.071**	−0.110***	−0.070**	0.014	−0.180***	1					
11.Indir	0.053*	−0.055**	−0.142***	0.061**	–0.029	0.163***	−0.128***	0.016	0.185***	0.065**	1				
12.Gender	–0.023	0.072**	–0.015	0.118***	0.068**	–0.036	0.053*	0.018	0.049*	0.090***	−0.102***	1			
13.Age	−0.054*	−0.053*	−0.072**	0.060**	0.036	0.075***	–0.004	0.091***	0.115***	0.057**	0.095***	0.050*	1		
14.Degree	0.099***	–0.021	0.020	–0.003	–0.021	0.008	−0.133***	–0.019	–0.042	–0.004	–0.034	–0.031	–0.002	1	
15.Oversee	0.036	0.090***	0.031	–0.0390	–0.035	–0.017	–0.026	0.043	–0.043	–0.016	–0.023	−0.134***	0.041	0.159***	1

*p < 0.1, **p < 0.05, ***p < 0.01.

### Regression Analysis

#### CEO Narcissism and R&D Investment

As shown in Table 4, Model 1 and Model 2 examine the results of testing the degree of CEO narcissism on firms’ investment in innovation. Model 1 analyzes the effects of each control variable on firms’ R&D investment. The results show that return on total assets, cash holdings, firm size, and dual employment are significantly and positively related to R&D investment, while gearing ratio, book-to-market ratio, and CEO gender are significantly and negatively related to R&D investment. Model 2 adds the independent variable CEO narcissism to model 1, and it can be seen that the regression coefficient of CEO narcissism is −0.008, which is prominent at the 1% level. The study proves that CEO narcissism has a negative effect on R&D investment, and the short-sighted cognitive bias makes narcissistic CEOs extraordinarily sensitive to the short-term returns that investment decisions can bring to them personally, and more inclined to make aggressive investment decisions such as foreign mergers and acquisitions, rather than investing in R&D activities with long payback periods. Hypothesis 1 is thus supported.

**TABLE 4:** Regression analysis results.

	RD						
	Model 1	Model 2	Model 3	Model 4	Model 5	Model 6	Model 7
**Control variables**							
Roa	0.017**	0.015*	0.014*	0.096***	−0.005	−0.047**	0.022**
	(0.009)	(0.009)	(0.009)	(0.018)	(0.010)	(0.023)	(0.009)
Lev	−0.028***	−0.028***	−0.028***	0.002	−0.034***	−0.017*	−0.033***
	(0.005)	(0.005)	(0.005)	(0.008)	(0.006)	(0.009)	(0.006)
MTB	−0.029***	−0.029***	−0.028***	0.004	−0.035***	0.011	−0.037***
	(0.005)	(0.005)	(0.005)	(0.007)	(0.006)	(0.010)	(0.005)
CF	0.020**	0.020**	0.021**	−0.020	0.035***	0.051**	0.009
	(0.009)	(0.009)	(0.009)	(0.013)	(0.011)	(0.020)	(0.010)
Size	0.001**	0.001**	0.001	−0.003**	0.001	−0.003***	0.004***
	(0.001)	(0.001)	(0.001)	(0.001)	(0.002)	(0.001)	(0.001)
Gender	−0.005**	−0.004	−0.004*	−0.000	−0.004		−0.003
	(0.002)	(0.002)	(0.002)	(0.004)	(0.003)		(0.002)
Age	−0.000	−0.000	−0.000	0.000	−0.000**	−0.001***	0.000
	(0.000)	(0.000)	(0.000)	(0.000)	(0.000)	(0.000)	0.000
Degree	0.000	0.000	0.000	−0.001	0.001**	0.003***	−0.000
	(0.000)	(0.000)	(0.000)	(0.001)	(0.001)	(0.001)	(0.001)
Oversee	0.003	0.002	0.002	−0.002	0.000	−0.007**	0.005***
	(0.002)	(0.002)	(0.002)	(0.003)	(0.002)	(0.003)	(0.002)
Dual	0.004***	0.004***	0.003***	0.012***	0.001	0.003	0.004***
	(0.001)	(0.001)	(0.001)	(0.002)	(0.001)	(0.003)	(0.001)
Indir	0.010	0.010	0.007	0.039***	−0.007	0.049***	0.001
	(0.009)	(0.009)	(0.009)	(0.013)	(0.013)	(0.016)	(0.011)
**Independent variable**							
Nar		−0.008***	−0.008***	0.012*	−0.011**	0.001	−0.009***
		(0.003)	(0.003)	(0.007)	(0.004)	(0.005)	(0.003)
**Moderator variable**							
FC			−0.002	−0.007	0.003		
			(0.004)	(0.007)	(0.006)		
**Interaction term**							
Nar × FC			0.021**	0.044**	0.021		
			(0.009)	(0.020)	(0.016)		
Year	Control	Control	Control	Control	Control	Control	Control
Industry	Control	Control	Control	Control	Control	Control	Control
*N*	1282	1282	1282	427	855	260	1022
*R* ^2^	0.426	0.431	0.433	0.754	0.405	0.737	0.429

*p < 0.1, **p < 0.05, ***p < 0.01.

#### The Moderating Effect of Financing Constraints

Model 3 reports the moderating effect of corporate financing constraints on the relationship between CEO narcissism and corporate R&D investment. The results show that the coefficient of CEO narcissism is significantly negative at the 1% level, and the interaction term coefficient is significantly positive at the 5% level. This indicates that the inhibitory effect of CEO narcissism on R&D investment weakens as the degree of financing constraints increases. Corporate financing constraints play a negative moderating role between CEO narcissism and R&D investment. Models 4 and 5 report the moderating effect of financing constraints in the large firm group and the small firm group. In the small firm group, the coefficient of CEO narcissism is significantly negative, and the coefficient of the interaction term does not pass the significance test. In the large firm group, the coefficient of CEO narcissism is significantly positive at the 10% level, and the interaction term coefficient is significantly positive at the 5% level. Thus, hypotheses 2a and 2b are supported.

#### The Moderating Effect of the Nature of Ownership

Model 6 and Model 7 are to test whether CEO narcissism and R&D investment are affected by the nature of firm ownership. The sample is divided into state-owned enterprise groups and non-state-owned enterprise groups, and regression analysis is conducted separately. In the state-owned enterprise group, the regression coefficient of CEO narcissism is 0.001, which does not pass the significance test, and in the non-state-owned enterprise group, the regression coefficient of CEO narcissism is −0.009, which is significant at the 1% level. The results indicate that CEO narcissism significantly inhibits the level of corporate innovation investment in the non-SOE group. As analyzed above, compared with SOEs, private enterprises have restricted and unstable sources of capital and lack certain government connections, which means they are less supportive than SOEs in terms of financing ability and operational security in terms of policy support. Under the innate environmental constraints, CEOs of non-SOEs with narcissism need to be more cautious about R&D investment and focus more on how to achieve rapid corporate growth in the short term and through M&A means. In contrast, narcissistic CEOs can acquire resources more rapidly, and narcissistic CEOs will reduce their firms’ innovation activities. Therefore, hypothesis 3 is tested.

#### Robustness Test

The CEO’s signature material in this paper comes from the IPO prospectus, and some may question the timeliness of the signature. However, according to the descriptive statistics of variables in Table 2, the average age of executives is 50 years old, which indicates that their personalities have become mature and stable. Narcissism is a personal character trait, so the degree of narcissism at this age is not easy to change. Therefore, we do not think that the signature statute of limitations will have an impact on the conclusion of this paper. However, to ensure the robustness of the results, we adopted the method of changing the sample size, limiting the sample to 3 years, and the results are shown in columns (1) to (5) of Table 5, where the regression results are consistent with the previous paper, and the conclusions of this paper are robust.

**TABLE 5:** Regression analysis results.

	RD						
	Model 1	Model 2	Model 3	Model 4	Model 5	Model 6	Model 7
**Control variables**							
Roa	0.004	0.003	−0.000	0.028	−0.004	−0.043	0.021
	(0.012)	(0.012)	(0.012)	(0.036)	(0.014)	(0.043)	(0.013)
Lev	−0.032***	−0.032***	−0.032***	0.009	−0.029***	−0.019	−0.029***
	(0.009)	(0.009)	(0.009)	(0.017)	(0.011)	(0.019)	(0.010)
MTB	−0.022***	−0.022***	−0.022***	0.029*	−0.020*	0.012	−0.028***
	(0.008)	(0.008)	(0.008)	(0.015)	(0.010)	(0.024)	(0.009)
CF	0.038**	0.037**	0.042**	0.036	0.034*	0.067	0.022
	(0.017)	(0.017)	(0.017)	(0.032)	(0.020)	(0.045)	(0.019)
Size	0.002	0.002	0.003*	−0.003	−0.003	−0.006**	0.002*
	(0.001)	(0.001)	(0.002)	(0.003)	(0.004)	(0.003)	(0.001)
Gender	−0.007*	−0.006	−0.006	−0.037***	−0.007*	0.000	−0.006
	(0.004)	(0.004)	(0.004)	(0.013)	(0.004)	(.)	(0.004)
Age	−0.000	−0.000	−0.000	0.000	−0.000	−0.001**	0.000
	(0.000)	(0.000)	(0.000)	(0.000)	(0.000)	(0.000)	(0.000)
Degree	0.001	0.000	0.001	−0.002	0.001	0.006**	−0.001
	(0.001)	(0.001)	(0.001)	(0.002)	(0.001)	(0.003)	(0.001)
Oversee	0.007*	0.006*	0.006*	−0.037***	0.007*	−0.005	0.008**
	(0.003)	(0.003)	(0.003)	(0.013)	(0.004)	(0.007)	(0.004)
Dual	0.003	0.002	0.002	0.013***	0.000	−0.004	0.004*
	(0.002)	(0.002)	(0.002)	(0.004)	(0.002)	(0.006)	(0.002)
Indir	0.002	0.000	−0.002	−0.014	−0.014	0.045	−0.018
	(0.017)	(0.017)	(0.017)	(0.028)	(0.022)	(0.034)	(0.020)
**Independent variable**							
Nar		−0.008*	−0.010**	0.042**	−0.005	0.015	−0.012**
		(0.005)	(0.005)	(0.021)	(0.007)	(0.011)	(0.005)
**Moderator variable**							
FC			0.008	−0.014	0.004		
			(0.008)	(0.020)	(0.011)		
**Interaction term**							
Nar × FC			0.025*	0.189***	−0.010		
			(0.016)	(0.072)	(0.025)		
Year	Control	Control	Control	Control	Control	Control	Control
Industry	Control	Control	Control	Control	Control	Control	Control
*N*	526	526	526	175	351	101	425
*R* ^2^	0.407	0.411	0.415	0.766	0.395	0.715	0.442

*p < 0.1, **p < 0.05, ***p < 0.01.

## Discussion

Although studies have explored the impact of CEO narcissism on the economic consequences of firms from different perspectives, there is little literature exploring corporate R&D investment from the perspective of financing constraints. The studies are imperfect, and most of them are based on scenarios in developed countries in Europe and the United States. In the context of building an innovative country in China, this paper analyzes the relationship between CEO narcissism and corporate R&D investment based on the data of A-share listed companies in Shanghai and Shenzhen from 2007 to 2020 and explores in depth the moderating mechanism of CEO narcissism affecting corporate R&D investment from two dimensions: financing constraints and the nature of corporate ownership. The difference in personality traits and its magnitude among CEOs leads to cognitive diversity, which can positively and negatively affect firms’ strategic decision processes and choices (Abatecola and Cristofaro, 2020). The results of the empirical study show that CEO narcissism significantly inhibits corporate R&D investment. Entrepreneurs are more aware of the chain of heuristic reasoning that is activated by their positive or negative affective disposition when facing the decisional situation (Cristofaro and Giannetti, 2021). Narcissistic leaders generally have vanity and a desire for attention, which makes them more eager to pursue scale and build a “business empire” through mergers and acquisitions at the expense of R&D investment (Raskin and Terry, 1988), which is consistent with our results. From the perspective of corporate financing, the inhibitory effect of CEO narcissism on R&D investment is weakened when companies face financing constraints. Smaller firms have more external financing premiums than larger firms (Gertler and Gilchrist, 1993). Financing constraints play a significant moderating role between CEO narcissism and corporate R&D investment in large firms compared to small firms. From the dimension of corporate ownership nature, the inhibitory effect of CEO narcissism on corporate R&D investment is more significant in non-state-owned companies compared with state-owned companies. This is due to the fact that SOEs have a rich source of external funding to provide reliable financial support for innovation, which in turn can reduce the risk of uncertainty due to the uncertainty of institutional and policy changes and promote innovation (Choi et al., 2011). This paper incorporates the personality trait of CEO narcissism into the research framework of corporate innovation, providing a new perspective for the study of the motivation of corporate innovation, extending the study of the economic consequences of CEO narcissism traits, and expanding the measurement of CEO narcissism by using CEO signature size as a proxy indicator of narcissism and selecting indicators with stable performance and fully controlled by executives themselves for effective measurement. By introducing two moderating variables, financing constraints and the nature of firm ownership, this paper complements and enriches the research perspective of contextual constraints on the decision-making behavior of narcissistic CEOs, providing new evidence to the literature on executive narcissism and R&D investment as well as enriching the literature on the economic consequences of executive narcissism.

## Conclusion and Limitation

This study extends the existing theory of higher echelons to provide more effective guidance in practice. First, this paper finds that executive narcissistic traits are a major factor influencing corporate decision-making (Yang et al., 2021; Li et al., 2022), and narcissistic CEOs do not tend to carry out innovative activities, especially in private firms where the negative effect of CEO narcissism is more prominent, so the findings of this paper can provide practical lessons for firms hiring executives. It is true that a good professional manager not only reflects his or her ability to handle all aspects of the company but also how his or her personal characteristics and preferences affect his or her company and determine its future direction, success, or failure. The CEOs should not be examined solely on the basis of their personal capabilities; the narcissistic traits of the CEO should match the company’s image, positioning, and strategic choice preferences. Therefore, we should have a comprehensive and objective understanding of the benefits and risks that CEO narcissism brings to enterprises. When selecting senior executives, enterprises should consider their psychological quality and personality traits and examine the level of psychological factors and personality traits of candidates through psychological tests to see whether candidates have highly narcissistic personality traits so as to avoid the inhibition of R&D investment caused by CEO narcissism from the source. In the context of China’s “Made in China 2025” strategy, the findings of this paper can provide an important theoretical reference for the selection of CEOs and explore an effective path for China to transform into an innovative country as soon as possible. Second, enterprises should establish supervision and incentive mechanisms for the management of executives. For the supervision mechanism, enterprises should establish a model of accountable decision-making. In this paper, we found that narcissistic CEOs are reluctant to implement R&D investment strategies, and this choice is most likely to be the CEO’s decision to make excessive M&A foreign expansion strategies to satisfy narcissistic needs, which is an important self-interest performance of narcissistic CEOs (Rosenthal and Pittinsky, 2006). Therefore, establishing accountability can, to a certain extent, avoid the over-expansion behavior of narcissistic CEOs and neglect R&D innovation behavior, thus reducing the negative impact of narcissistic CEOs on the firm. Additionally, for CEOs with narcissistic traits, companies can implement a combination of explicit material incentives and implicit spiritual incentives. Explicit material incentives include performance pay and stock incentives, etc. In terms of implicit spiritual incentives, narcissistic CEOs pay extra attention to their own image and reputation, and enterprises can help CEOs to carry out word-of-mouth publicity in the public and industry and give certain job promotions. Third, enterprises can moderately adjust the financing ability and make reasonable use of the negative regulatory effect of narcissistic CEO on enterprise R&D investment. The financing constraint is a key variable that affects the R&D behavior of an organization, and it is a factor that cannot be ignored in the strategic decision-making process of CEOs. The degree of financing constraints varies by firm size and small firms are more financially constrained than large firms (Gupta et al., 2021). This paper shows that the inhibitory effect of narcissistic CEOs on R&D investment is weakened when firms face financing constraints, and the moderating effect of financing constraints is more prominent in large firms. However, excessive financing constraints may cause firms to fall into financial distress. Therefore, firms need to set a reasonable capital structure to support their R&D activities financially. Fourth, this paper compares the effects of CEO narcissism on corporate R&D investment in state-owned and non-state-owned enterprises. In state-owned enterprises, the performance of CEO narcissistic traits is less prominent than in non-state-owned enterprises because management decision-making power is restricted. While state-owned enterprises have strong capital and abundant research resources, enterprises can formulate salary and promotion standards linked to performance to improve resource utilization efficiency and governance effects. Meanwhile, the government increases support to private enterprises to alleviate the financing difficulties of SMEs and increase the investment in R&D activities in state-owned and non-state-owned enterprises.

The study described in this paper may have several shortcomings: First, the CEO narcissism indicator measure. The CEO narcissism signature used in this paper is derived from IPO prospectuses of listed companies. Although CEO narcissism is a relatively stable personality trait, CEO narcissism trait is also affected by its own factors and the internal and external environment, which can produce subtle changes. Moreover, Chinese handwriting is highly variable, such as the rigidity, flexibility, and neatness of scribbles, which can reflect the personality psychology of the writer. Therefore, in subsequent research, combining the handwriting research of Chinese characters with corporate behavior, it is believed that more research results with Chinese characteristics can be excavated. Second, the influence mechanism. In this paper, we mainly explore the influence mechanism between CEO narcissism and corporate innovation investment from two levels: corporate financing and ownership nature, but due to the limitation of the study, there may be other influence mechanisms that exist, and more paths and boundaries can be considered to enrich the existing theoretical results.

## Data Availability Statement

The original contributions presented in the study are included in the article/Supplementary Material, further inquiries can be directed to the corresponding author/s.

## Author Contributions

HL and YM: writing the manuscript. LW: providing and revised the advice. All authors contributed to the article and approved the submitted version.

## Conflict of Interest

The authors declare that the research was conducted in the absence of any commercial or financial relationships that could be construed as a potential conflict of interest.

## Publisher’s Note

All claims expressed in this article are solely those of the authors and do not necessarily represent those of their affiliated organizations, or those of the publisher, the editors and the reviewers. Any product that may be evaluated in this article, or claim that may be made by its manufacturer, is not guaranteed or endorsed by the publisher.
